# Supplementary material for: The BIN1 rs744373 SNP is associated with increased tau-PET levels and impaired memory
Source: Nat Commun. 2019 Apr 16;10:1766. doi: 10.1038/s41467-019-09564-5 (PMC6467911; doi:10.1038/s41467-019-09564-5)
Supplement: Supplementary file 2 — Reporting Summary [file 41467_2019_9564_MOESM2_ESM.pdf]

nature research

Corresponding author(s): Michael Ewers

Reporting Summary

Nature Research wishes to improve the reproducibility of the work that we publish. This form provides structure for consistency and transparency in reporting. For further information on Nature Research policies, see Authors & Referees and the Editorial Policy Checklist.

Statistical parameters

When statistical analyses are reported, confirm that the following items are present in the relevant location (e.g. figure legend, table legend, main text, or Methods section).

n/a	Confirmed	
☐	☒	The exact sample size (*n*) for each experimental group/condition, given as a discrete number and unit of measurement
☐	☒	An indication of whether measurements were taken from distinct samples or whether the same sample was measured repeatedly
☐	☒	The statistical test(s) used AND whether they are one- or two-sided<br>*Only common tests should be described solely by name; describe more complex techniques in the Methods section.*
☐	☒	A description of all covariates tested
☐	☒	A description of any assumptions or corrections, such as tests of normality and adjustment for multiple comparisons
☐	☒	A full description of the statistics including central tendency (e.g. means) or other basic estimates (e.g. regression coefficient) AND variation (e.g. standard deviation) or associated estimates of uncertainty (e.g. confidence intervals)
☐	☒	For null hypothesis testing, the test statistic (e.g. *F*, *t*, *r*) with confidence intervals, effect sizes, degrees of freedom and *P* value noted<br>*Give P values as exact values whenever suitable.*
☒	☐	For Bayesian analysis, information on the choice of priors and Markov chain Monte Carlo settings
☒	☐	For hierarchical and complex designs, identification of the appropriate level for tests and full reporting of outcomes
☐	☒	Estimates of effect sizes (e.g. Cohen's *d*, Pearson's *r*), indicating how they were calculated
☐	☒	Clearly defined error bars<br>*State explicitly what error bars represent (e.g. SD, SE, CI)*

Our web collection on statistics for biologists may be useful.

Software and code

Policy information about availability of computer code

Data collection	Data used in preparation of this manuscript were obtained from the ADNI database (adni. loni.usc.edu).
Data analysis	All data was analyzed using RStudio statistical software, Version 1.1.414

For manuscripts utilizing custom algorithms or software that are central to the research but not yet described in published literature, software must be made available to editors/reviewers upon request. We strongly encourage code deposition in a community repository (e.g. GitHub). See the Nature Research guidelines for submitting code & software for further information.

Data

Policy information about availability of data

All manuscripts must include a data availability statement. This statement should provide the following information, where applicable:

- Accession codes, unique identifiers, or web links for publicly available datasets
- A list of figures that have associated raw data
- A description of any restrictions on data availability

All imaging data was downloaded from the ADNI loni image archive (https://ida.loni.usc.edu).

Field-specific reporting

Please select the best fit for your research. If you are not sure, read the appropriate sections before making your selection.

- [x] Life sciences
- [ ] Behavioural & social sciences
- [ ] Ecological, evolutionary & environmental sciences

For a reference copy of the document with all sections, see nature.com/authors/policies/ReportingSummary-flat.pdf

Life sciences study design

All studies must disclose on these points even when the disclosure is negative.

Sample size	We included 89 participants from ADNI phase 3 (ClinicalTrials.gov ID: NCT02854033) in whom 18F-AV1451 tau-PET was obtained. 18F-AV1451 PET was added only in phase 3 of ADNI, and is thus only available in a smaller subset of the large ADNI cohort. The current set of 89 subjects resulted from inclusion criteria of the availability of T1-weighted MRI, 18F-AV45 amyloid-PET, cognitive and GWAS data in addition to 18F-AV1451 tau-PET. All imaging modalities had to be obtained at the same study visit
Data exclusions	No data was excluded
Replication	No replicability analysis was performed in the current study
Randomization	Allocation to groups was based on genetics (BIN1 rs744373 allele) and diagnosis, so no randomization was done. BIN1 rs744373 allele distribution (GG/GA/AA=8/32/49) did not deviate from Hardy-Weinberg equilibrium (p=0.422). There were no differences in baseline demographics (age, gender, education) between cognitively normal vs. mild cognitively impaired subjects or between BIN1 rs744373 risk-allele vs. normal allele carriers.
Blinding	Blinding was not possible during analysis

Reporting for specific materials, systems and methods

Materials & experimental systems

n/a	Involved in the study
[x]	[ ] Unique biological materials
[x]	[ ] Antibodies
[x]	[ ] Eukaryotic cell lines
[x]	[ ] Palaeontology
[x]	[ ] Animals and other organisms
[ ]	[x] Human research participants

Methods

n/a	Involved in the study
[x]	[ ] ChIP-seq
[x]	[ ] Flow cytometry
[ ]	[x] MRI-based neuroimaging

Human research participants

Policy information about studies involving human research participants

Population characteristics	For the current study, we analyzed data from 89 participants of the ADNI cohort, including 49 cognitively normal (CN) and 40 mild cognitively impaired (MCI) subjects. We found 22 CN subjects and 18 MCI subjects to carry at least one copy of the BIN1 rs744373 G-allele which confers higher risk of AD dementia. There were no differences in baseline demographics (age, gender, education) between CN vs. MCI or between BIN1 rs744373 risk-allele vs. normal allele carriers. In total 48 subjects (24 CN &, 24 MCI) showed abnormally elevated amyloid levels as determined via AV45 PET (i.e. global standardized uptake value ratio (SUVR)>1.11).
Recruitment	All subjects were recruited within the Alzheimer's Disease Neuroimaging Initiative (ADNI, see http://adni.loni.usc.edu/adni-3/). The authors of the study were not involved in subject recruitment

Magnetic resonance imaging

Experimental design

Design type	Structural MRI, no task was performed

Design specifications	n/a
Behavioral performance measures	n/a

Acquisition

Imaging type(s)	structural
Field strength	3
Sequence & imaging parameters	Field of View: 208x240x256mm; 1x1x1mm Voxel Resolution; TE=min full echo; TR=2300; TI=900; 2X accelerated image acquisition
Area of acquisition	Whole brain scan was used

Diffusion MRI ☐ Used ☒ Not used

Preprocessing

Preprocessing software	Preprocessing was conducted with Freesurfer 5.3, SPM12 and in-house matlab scripts
Normalization	Using SPMs high-dimensional DARTEL warping algorithm, we estimated subject-specific flow-fields to non-linearly transform all grey matter, white matter and cerebrospinal fluid maps to a sample specific template that was determined in an iterative procedure. Using affine transformation, this sample-specific template was subsequently normalized to Montreal Neurological Institute (MNI) standard space. Next, subject-specific AV45 amyloid-PET images were co-registered to the corresponding high-resolution T1 image and subsequently DARTEL warped to MNI standard space.
Normalization template	MNI
Noise and artifact removal	No functional MRI was used, so no artifact removal was performed
Volume censoring	n/a

Statistical modeling & inference

Model type and settings	Univariate
Effect(s) tested	For our main analysis, we tested whether BIN1 rs744373 risk-allele carriage was associated with increased AV1451 tau-PET uptake. To this end, we applied ANCOVAs to test whether presence of the BIN1 rs744373 risk allele had an effect on global or regional (i.e. Braak-stage) AV1451 tau-PET SUVRs, controlling for age, gender, education, diagnosis and ApoE [illegible] 4 carrier status and grey matter volume of the respective tau ROI. To assess any effects of the BIN1 rs744373 SNP on amyloid, we tested the same models this time using global or regional (i.e. amyloid-stages) AV45 amyloid-PET SUVRs. Lastly, we tested whether BIN1 rs744373 risk allele carriage was associated with higher AV1451 tau-PET uptake independent of amyloid. To this end, we conducted linear regression with global AV1451 tau-PET SUVR as a dependent variable and global AV45 amyloid-PET SUVR and BIN1 rs744373 status as independent variables controlling for age, gender, education, ApoE e4 carrier status and diagnosis.

Specify type of analysis: ☐ Whole brain ☒ ROI-based ☐ Both

Anatomical location(s)	Anatomical locations were determined on pre-established braak (Schöll et al., Neuron, 2017) and amyloid staging ROIs (Grothe et al., Neurology, 2017)
Statistic type for inference (See Eklund et al. 2016)	n/a
Correction	Bonferroni correction

Models & analysis

n/a	Involved in the study
☒	☐ Functional and/or effective connectivity
☒	☐ Graph analysis
☒	☐ Multivariate modeling or predictive analysis
